# Porous NaTi_2_(PO_4_)_3_ Nanocubes Anchored on Porous Carbon Nanosheets for High Performance Sodium-Ion Batteries

**DOI:** 10.3389/fchem.2018.00396

**Published:** 2018-09-19

**Authors:** Ziqi Wang, Jiaojiao Liang, Kai Fan, Xiaodi Liu, Caiyun Wang, Jianmin Ma

**Affiliations:** ^1^School of Physics and Electronics, Hunan University, Changsha, China; ^2^College of Chemistry and Pharmaceutical Engineering, Nanyang Normal University, Nanyang, China; ^3^ARC Centre of Excellence for Electromaterials Science, Intelligent Polymer Research Institute, AIIM Facility, University of Wollongong, North Wollongong, NSW, Australia; ^4^Institute of Advanced Electrochemical Energy, Xi'an University of Technology, Xi'an, China

**Keywords:** NaTi_2_(PO_4_)_3_, nanocubes, carbon nanosheets, anode, sodium-ion batteries

## Abstract

NaTi_2_(PO_4_)_3_ has attracted great interest as anode material for sodium ion batteries owing to its open three-dimensional framework structure and limited volume changes during the charge and discharge process. However, the poor intrinsic electronic conductivity of NaTi_2_(PO_4_)_3_ needs to be improved for high rate capability. In this work, porous NaTi_2_(PO_4_)_3_ nanocubes anchored on porous carbon nanosheets (NaTi_2_(PO_4_)_3_/C) are designed and developed. This material exhibits a large discharge capacity and good rate capacity including a first discharge capacity of 485 mAh g^−1^ at a current density of 0.1 A g^−1^, and 98 mAh g^−1^ retained at a high rate of 4 A g^−1^ even after 2,000 cycles. These results suggest that NaTi_2_(PO_4_)_3_/C is a promising anode material for sodium-ion batteries.

## Introduction

Sodium-ion batteries (SIBs), as an alternative energy storage system for lithium-ion batteries (LIBs), have attracted increasing attention due to their low cost and the abundant resource of sodium (Gao et al., 2017; Cui et al., 2018; Liang et al., 2018c). The electrochemical performance of SIBs is closely related to the properties of electrode materials, especially anode materials (Chen et al., 2018; Fan et al., 2018; Hu A. J. et al., 2018; Liang et al., 2018b,d; Wan et al., 2018; Wei et al., 2018). Recently, Na super ion conductor (NASICON) type NaTi_2_(PO_4_)_3_ has been considered as one of promising anode materials for SIBs owing to its “zero-stress” three-dimensional (3D) framework, high Na^+^ conductivity, and good thermal stability (Kabbour et al., 2011; Wu et al., 2013; Sun et al., 2016; Ye et al., 2017).

However, the poor intrinsic electrical conductivity of NTP leads to poor rate capability (Pang et al., 2014b; Roh et al., 2017). To improve the Na^+^ ions insertion-extraction kinetics, two common approaches used include synthesis of various nanostructures and fabrication of carbon composites. Morphology control of NaTi_2_(PO_4_)_3_ has been applied to realizeexcellent electrochemical performance. Different nanostructures such as hollow nanocubes, nanoparticles, and hierarchical microspheres have been demonstrated (Wu et al., 2015; Fang et al., 2016; Ye et al., 2017). Among them, porous structures have gained great attention owing to the afforded large surface areas and improved kinetics (Dirican et al., 2015; Zhang et al., 2017; Zhao et al., 2017; Zhou et al., 2017). Moreover, the electronic conductivity of anodes can be largely enhanced by hybridizing them with conductive materials. For example, the coating of carbon or graphene on NaTi_2_(PO_4_)_3_ micro/nanostructures can effectively improve their properties and higher quality of the conductive materials could result in better electrochemical performance. Nevertheless, the contents of carbon or graphene in the previously reported composites were only 3.4–6.8 wt% (Pang et al., 2014b; Fang et al., 2016; Geng et al., 2017; Hu Q. et al., 2018; Liang et al., 2018a). Thus, to obtain better electrochemical properties, the contents of conduction materials should be increased. It has been found that the embedding of anode materials in carbon/graphene matrixes can realize high content of conductive carbon materials for enhanced electrochemical properties (Fu et al., 2015; Guo et al., 2015; Choi et al., 2016; Sun et al., 2016). Motivated by the above potentials, we have prepared porous NaTi_2_(PO_4_)_3_ nanocubes anchored on porous carbon nanosheets (NaTi_2_(PO_4_)_3_/C) through ultrasonic treatment. To the best of our knowledge, it is the first report that NaTi_2_(PO_4_)_3_ nanocubes with porous structures have been embedded in a porous carbon matrix. This NaTi_2_(PO_4_)_3_/C material exhibited a high discharge capacity, good rate performance, and excellent long-time cycling stability.

## Experimental section

### Synthesis of NaTi_2_(PO_4_)_3_/C

The synthesis of porous carbon nanosheets was firstly conducted from the uniform mixture of Zn(CH_3_COO)_2_·2H_2_O (5 g) and oleic acid (5 g) in an agate mortar for 30 min. Then the above mixture was transferred into a tubular furnace and calcined at 700°C for 2 h with a ramping rate of 2°C min^−1^ in Ar atmosphere to form ZnO/C slices. The ZnO/C slices were washed using 6 mol L^−1^ aqueous HCl solution to form porous carbon nanosheets. These carbon nanosheets were washed by deionized water and absolute ethanol, then dried in vacuum at 50°C for 8 h. Similar process was used to prepare the MnO/graphene composite using oleic acid as carbon sources (Guo et al., 2015).

The synthesis of NaTi_2_(PO_4_)_3_ nanocubes was conducted following the reported procedures (Wu et al., 2015). Briefly, sodium acetate (0.16 g) was added in a mixed solvent glacial acetic acid (0.7 mL), phosphoric acid (4 ml) and ethylene glycol (25 ml), followed by the addition of tetrabutyl titanate (1.36 g). The resultant mixture was heated at 180°C for 12 h. Finally, the white precipitate NaTi_2_(PO_4_)_3_ was obtained.

For synthesizing NaTi_2_(PO_4_)_3_/C, 0.16 g precursor NaTi_2_(PO_4_)_3_, 0.04 g porous carbon nanosheets, and 3.6 g cetyltrimethylammonium bromide (CTAB) were added into 30 mL absolute ethanol. After being stirred for 2 h and ultrasonically dispersed for 2 h, the precursor NaTi_2_(PO_4_)_3_/C was collected by centrifugation, washed with deionized water and anhydrous ethanol, and dried at 60°C for 12 h. Subsequently, the precursor NaTi_2_(PO_4_)_3_/C was further calcined at 700°C for 2 h with a ramping rate of 2°C min^−1^ in Ar atmosphere to form NaTi_2_(PO_4_)_3_/C. For comparison, porous NaTi_2_(PO_4_)_3_ cubes were prepared after annealing without the porous carbon nanosheets.

### Characterizations

Rigaku D/max-2500 X-ray diffractometer (Cu Kα, λ = 1.54056 Å) was used to investigate the crystal structures. The morphology and nanostructure were observed by Hitachi S4800 scanning electron microscopy and JEOL 2010 transmission electron microscopy. The Brunauer-Emmett-Teller special (BET) surface area and pore size were tested at 77 K on a Nova 2000e volumetric adsorption analyzer. The thermogravimetric (TG) analysis was performed with a WCT-1D instrument over a range of 30–800°C at a heating rate of 10°C·min^−1^ in air atmosphere.

### Electrochemical measurements

Active materials, acetylene black and carboxymethylcellulose sodium with a weight ratio of 80:10:10 were uniformly mixed, and the obtained slurry was coated on Cu foil. Then, the electrodes were assembled into CR2025 coin cell in the glove box. The glass microfiber filter membrane (Whatman, grade GF/A) was used as the separator. Metallic sodium film was used as counter/reference electrodes. The electrolyte was 1 mol L^−1^ NaClO_4_ dissolved in a mixture of ethylene carbonate and diethyl carbonate (1:1 vol%) with 5 wt% fluoroethylene carbonate. Galvanostatic tests were evaluated by Neware Battery Testing System. Cyclic voltammetry (CV) tests and impedance measurement were carried out on a CHI660C Electrochemical Workstation.

## Results and discussion

**Figure 1:**
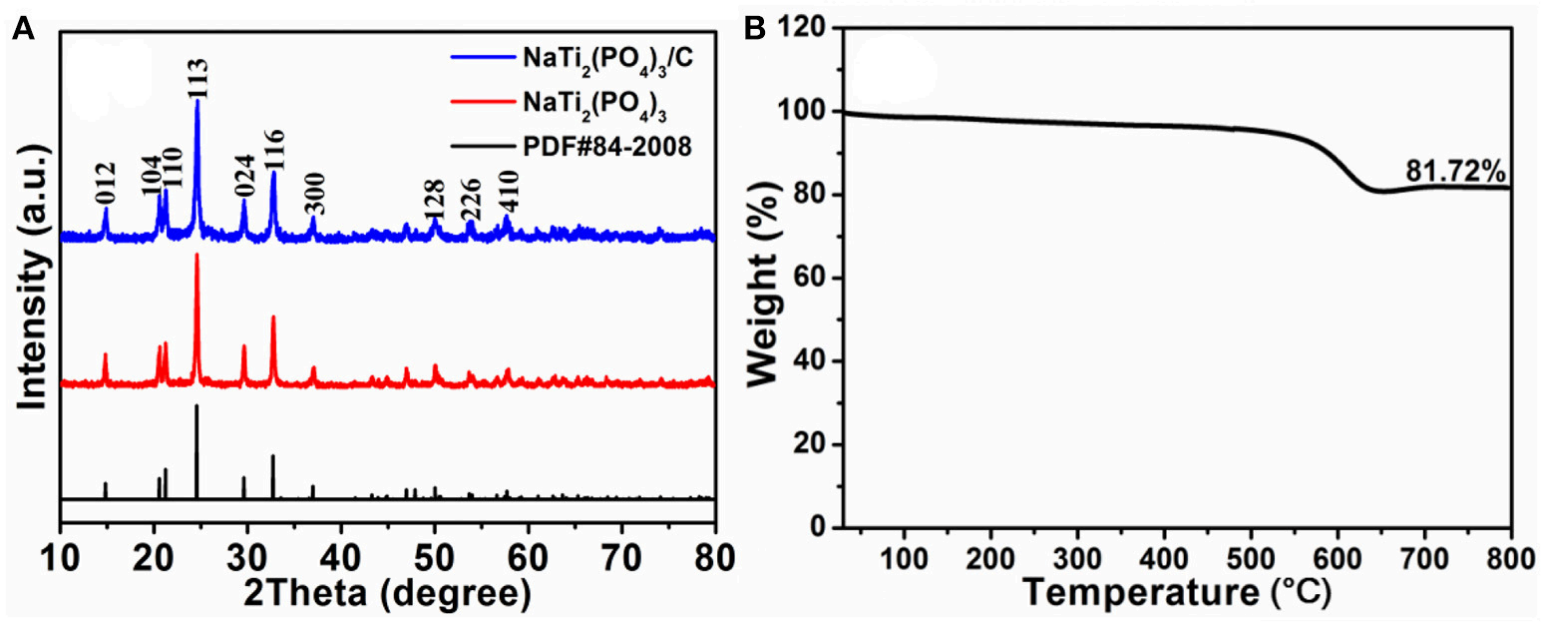
**(A)** XRD patterns of NaTi_2_(PO_4_)_3_ and NaTi_2_(PO_4_)_3_/C; **(B)** TG curve of NaTi_2_(PO_4_)_3_/C.

The phase of NaTi_2_(PO_4_)_3_ and NaTi_2_(PO_4_)_3_/C were confirmed by XRD, as shown in Figure 1A. All diffraction peaks are in accordance with the standard pattern of NaTi_2_(PO_4_)_3_ (JCPDS No. 84-2008). No peaks for impurities can be detected, suggesting the high purity of these two samples. Moreover, the diffraction peaks of carbon material is not clearly discerned due to the sharp and strong diffraction of NaTi_2_(PO_4_)_3_, implying the high crystalline nature. In addition, according to the TGA curve of NaTi_2_(PO_4_)_3_ (Figure 1B), the relative weight fraction of carbon for NaTi_2_(PO_4_)_3_/C was determined to be ~18.3%.

**Figure 2:**
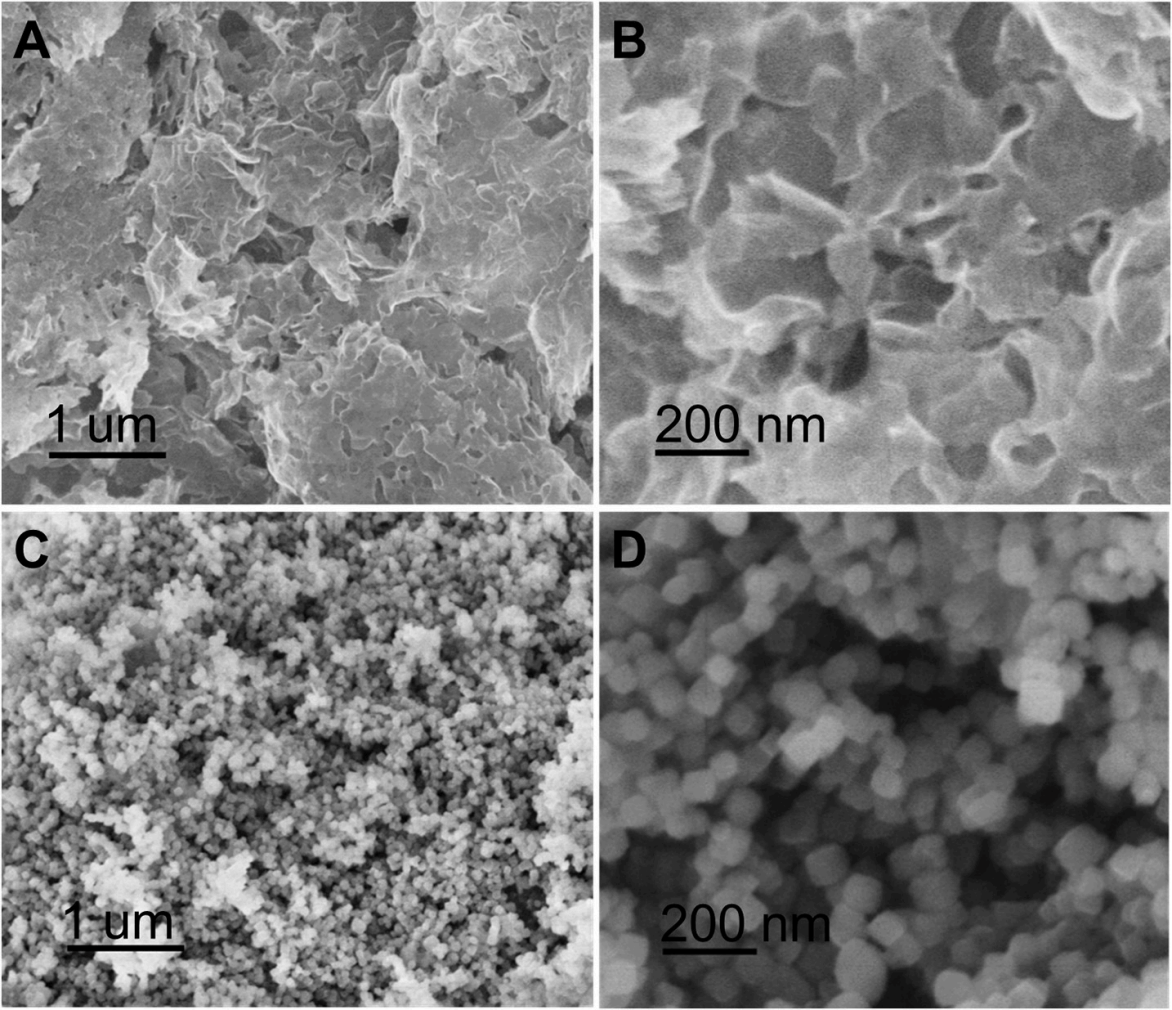
**(A)** SEM image and **(B)** high-resolution SEM image of porous carbon nanosheets; **(C)** SEM image and **(D)** high-magnification SEM image of NaTi_2_(PO_4_)_3_.

The morphology of porous carbon nanosheets, NaTi_2_(PO_4_)_3_, and NaTi_2_(PO_4_)_3_/C was characterized by SEM. Figure 2A shows the low-magnified SEM image of porous carbon nanosheets. It is clear that the sample is exclusively nanosheets with irregular morphologies. The high-resolution SEM image (Figure 2B) shows that the carbon nanosheets are curved and have an average thickness of ~10 nm. SEM images of NaTi_2_(PO_4_)_3_ (Figures 2C,D) show that the products have uniform cubic shapes and their sizes are in the range between 50 and 100 nm, similar with the results reported in literatures (Liang et al., 2018a).

**Figure 3:**
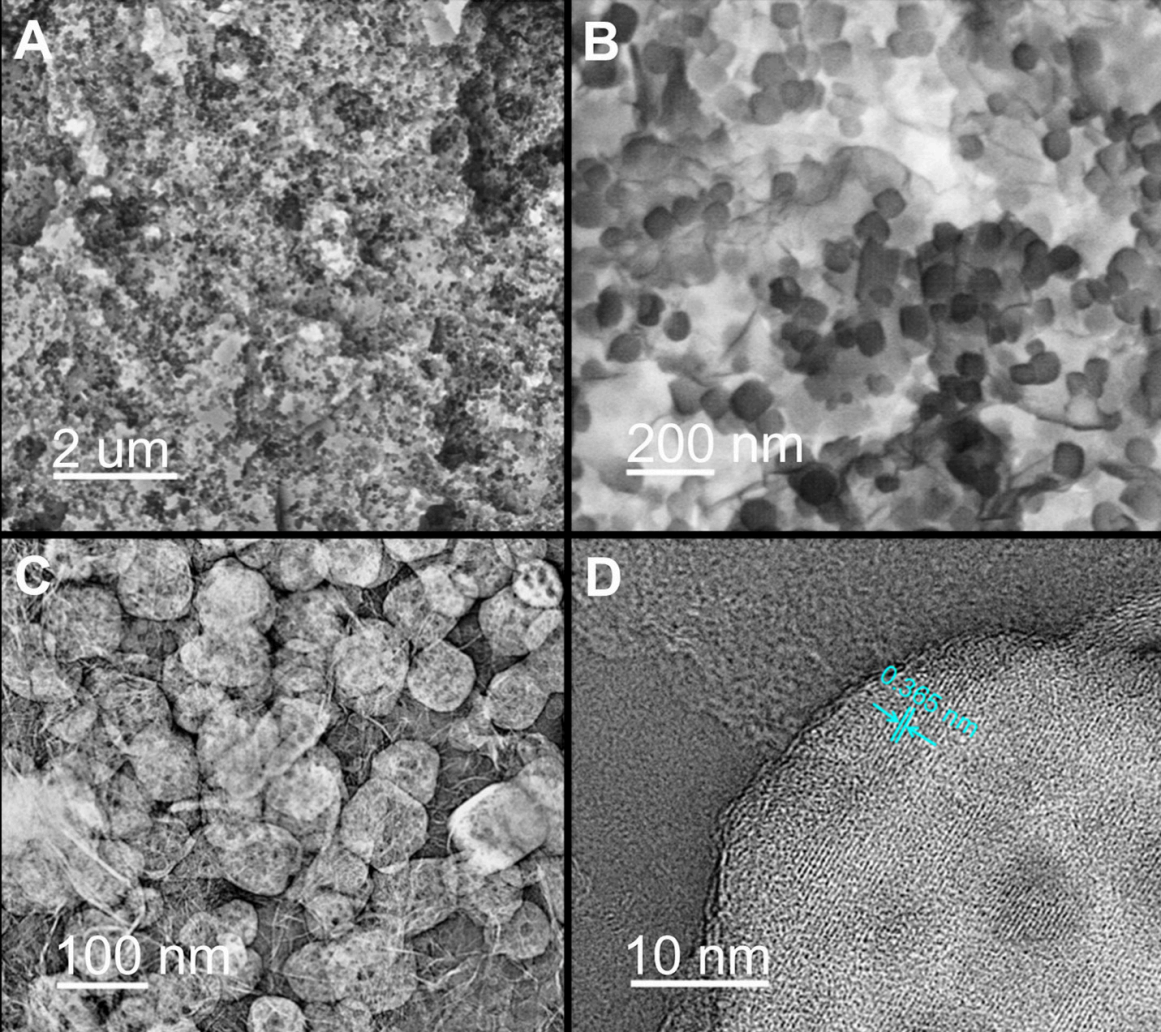
**(A)** SEM image, **(B)** high-resolution SEM image, and **(C)** TEM image of NaTi_2_(PO_4_)_3_/C; **(D)** HR-TEM images of a typical NaTi_2_(PO_4_)_3_ particle anchored on carbon nanosheets.

It was observed that the NaTi_2_(PO_4_)_3_ were uniformly anchored on the porous carbon nanosheets for NaTi_2_(PO_4_)_3_/C sample (Figures 3A,B). The detailed structural characteristics of NaTi_2_(PO_4_)_3_/C was further investigated by TEM. The TEM image in Figure 3C illustrates that NaTi_2_(PO_4_)_3_ in a size range of 50 and 100 nm were scattered over the carbon nanosheets. This is consistent with the SEM results in Figure 3B. Notably, both nanocubes and carbon nanosheets have obvious porous structure, which is beneficial for the transport of Na^+^ (Wu et al., 2015). Additionally, the HR-TEM image of a representative nanocube (Figure 3D) implies that the interplanar spacing is *ca*. 0.365 nm, in good agreement with the (113) plane of NASICON-type phase (Ye et al., 2017).

**Figure 4:**
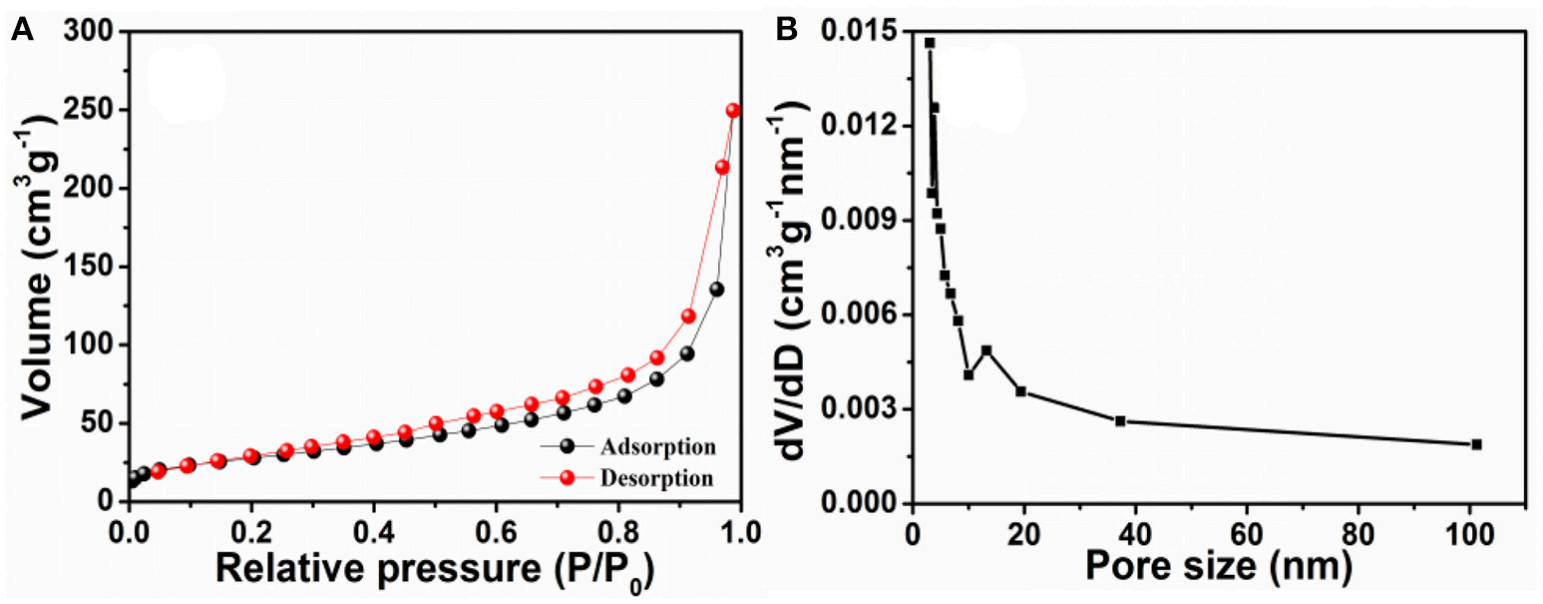
**(A)** N_2_ adsorption-desorption isotherm and **(B)** pore-size distribution curve of NaTi_2_(PO_4_)_3_/C.

BET analysis was performed to study the pore size and specific surface area of NaTi_2_(PO_4_)_3_/C. The Nitrogen adsorption-desorption isotherm of NaTi_2_(PO_4_)_3_/C (Figure 4A) reveals a type-IV isotherm with an obvious H1-type hysteretic loop in the range of 0.4–1.0 (*P*/*P*_0_), indicating that the products possess porous structures (Takashima et al., 2015). The BET analysis indicates that the specific surface area of NaTi_2_(PO_4_)_3_/C was *ca*. 103.1 m^2^ g^−1^. Moreover, as shown in Figure 4B, the sample possessed a broad pore-size distribution and the pore-size distribution maximum was centered at 15.4 nm. The large surface area and porous structure of NaTi_2_(PO_4_)_3_/C is beneficial to improve the sodium-ion storage properties (Wang H. et al., 2016; Wang G. et al., 2018).

**Figure 5:**
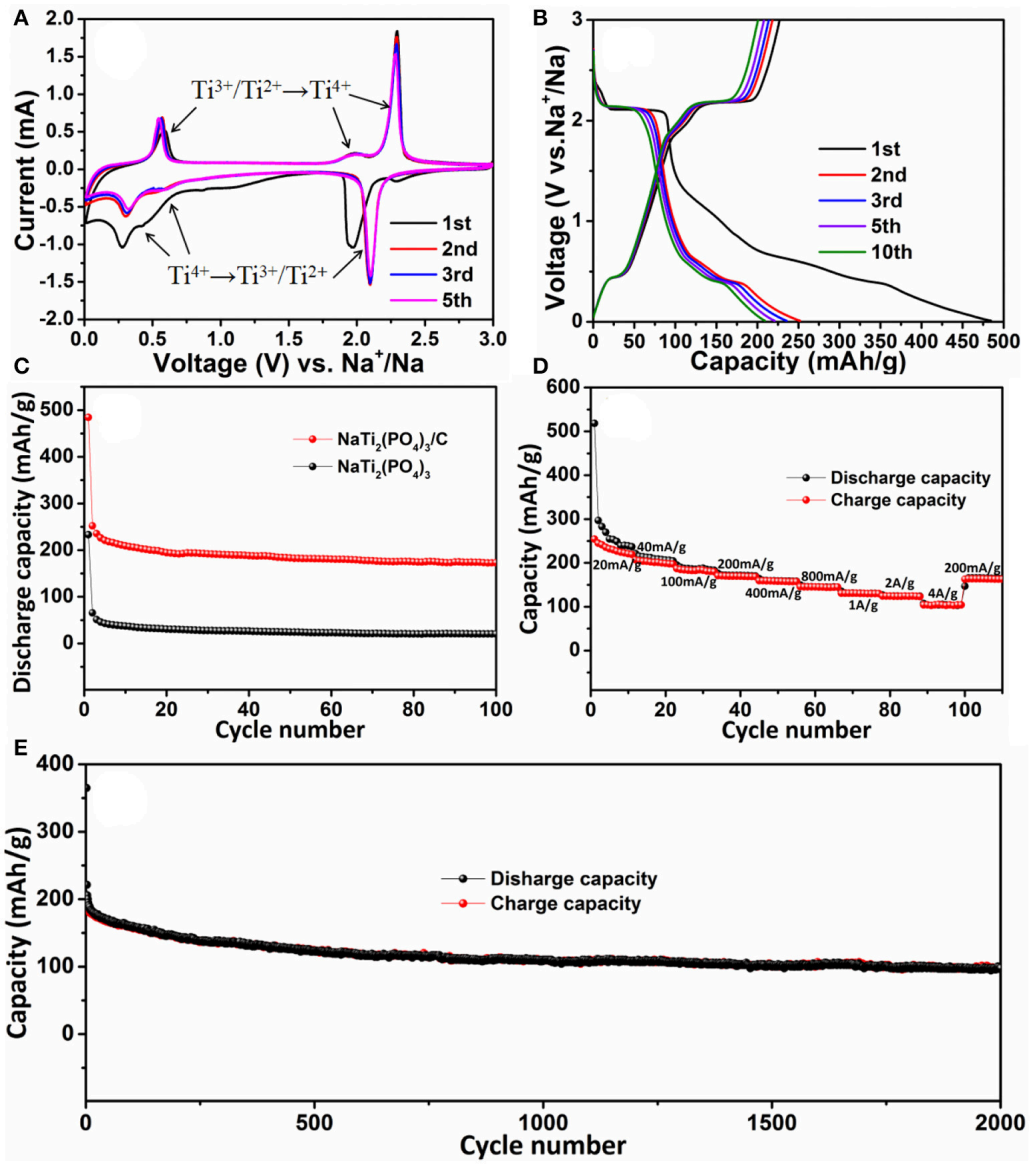
**(A)** Cyclic voltammogram curves of NaTi_2_(PO_4_)_3_/C for the initial five cycles in the voltage range of 0.01–3.0 V (vs. Na^+^/Na); **(B)** discharge-charge curves of NaTi_2_(PO_4_)_3_/C at a current density of 0.1 A g^−1^; **(C)** cycling performances of NaTi_2_(PO_4_)_3_/C and NaTi_2_(PO_4_)_3_ at 0.1 A g^−1^; **(D)** rate capacity of NaTi_2_(PO_4_)_3_/C; **(E)** long-term cycling performance of NaTi_2_(PO_4_)_3_/C at a high rate of 4 A g^−1^.

The electrochemical properties of NaTi_2_(PO_4_)_3_/C were studied as anode material for SIBs. The cyclic voltammogram (CV) of NaTi_2_(PO_4_)_3_/C at a scan rate of 0.1 mV s^−1^ was analyzed to investigate their redox kinetic properties. In Figure 5A, at the 1st cycle, a pair of redox peaks at 1.97/2.29 V can be attributed to conversion reaction of Ti^4+^/Ti^3+^ (Pang et al., 2014a; Fang et al., 2016; Ye et al., 2017). Moreover, another pair of cathodic/anodic peaks located at 0.27/0.57 V can be attributed to the redox reaction between Ti^3+^ and Ti^2+^ (Senguttuvan et al., 2013; Wang D. et al., 2016). That is, Ti^4+^ in the reduction process was firstly reduced to Ti^3+^ (NaTi_2_(PO_4_)_3_ + 2Na^+^ + 2e^−^ → Na_3_Ti_2_(PO_4_)_3_) and then formed into Ti^2+^ (Na_3_Ti_2_(PO_4_)_3_+ Na^+^ + e^−^ → Na_4_Ti_2_(PO_4_)_3_). In the following cycles, the anodic peaks shift to higher potentials (1.97 vs. 2.09 V; 0.27 vs. 0.30 V), which was probably caused by the stress/strain change, similar to other NASICON-type anodic materials (Li et al., 2014). More importantly, in the following 2nd, 3rd, and 5th cycles, two pairs of reduction/oxidation peaks almost remained unchanged, indicating the excellent reversibility.

Figure 5B showed the galvanostatic discharge-charge curves of NaTi_2_(PO_4_)_3_/C electrode in the voltage window between 0.01 and 3.0 V. The initial discharge capacity was 485 mAh g^−1^, which was higher than the theoretical capacity (133 mAh g^−1^). However, the initial charge capacity was 227 mAh g^−1^ with an unsatisfied Coulombic efficiency of 46.8%. Such a large capacity loss is mostly ascribed to the formation of solid electrolyte interface (SEI) layers for the existence of carbon substrates, as well as the decomposition of electrolyte (Hasegawa et al., 2016; Wang D. et al., 2016). On the contrary, the first discharge capacity of the NaTi_2_(PO_4_)_3_ electrode was only 229 mAh g^−1^ (Figure S1, Supporting Information). In the subsequent cycles, the NaTi_2_(PO_4_)_3_/C electrode possessed good cycle stability and excellent reversibility for Na^+^ ion insertion and extraction. For example, at the 5th and 10th cycles, the discharge capacity retained to be 221 and 203 mAh g^−1^ with the coulombic efficiency of 94 and 96%, respectively. Figure 5C displayed the cycling behavior of the NaTi_2_(PO_4_)_3_/C and NaTi_2_(PO_4_)_3_ electrodes at a current density of 0.1 A g^−1^. It can be seen thatafter 100 cycle NaTi_2_(PO_4_)_3_/C still delivered a discharge capacity of 172 mAh g^−1^, which was much larger than that of NaTi_2_(PO_4_)_3_ (20 mAh g^−1^). Accordingly, the NaTi_2_(PO_4_)_3_/C electrode exhibited a capacity retention of 69% (relative to the 2nd cycle), higher than that of NaTi_2_(PO_4_)_3_ (30%). In addition, the long-term cycling performance for the NaTi_2_(PO_4_)_3_/C electrode at a relatively high rate of 4 A g^−1^ was further studied. In Figure 5E, it can be clearly found that the Coulombic efficiency could exceed 98% since the 10th cycle, and the electrode can still maintain a discharge capacity of 98 mAh g^−1^ even after 2,000 cycles. All these results indicate that NaTi_2_(PO_4_)_3_/C afforded improved electrochemical stability compared with that of NaTi_2_(PO_4_)_3_.

Furthermore, the rate capability of NaTi_2_(PO_4_)_3_/C electrode was also investigated by increasing rate from 0.02 to 4 A g^−1^ and back to 0.2 A g^−1^. As illustrated in Figure 5D, the discharge capability of NaTi_2_(PO_4_)_3_/C was 280 mAh g^−1^ at 0.02 A g^−1^, and then it slowly decreased with the increasing current density. When the current density was reversed to 0.2 A g^−1^, a capacity of 164 mAh g^−1^ could be restored. Obviously, NaTi_2_(PO_4_)_3_/C has excellent rate capacity.

**Figure 6:**
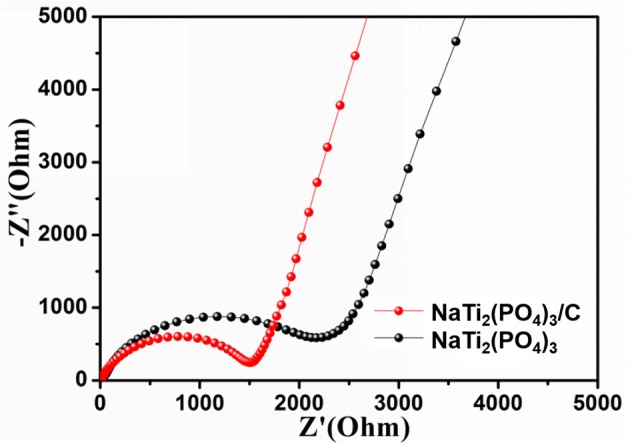
Nyquist plots of NaTi_2_(PO_4_)_3_/C and NaTi_2_(PO_4_)_3_.

Lastly, EIS measurements were carried out to further study the surface reaction activities of NaTi_2_(PO_4_)_3_/C and NaTi_2_(PO_4_)_3_. Before the EIS tests, the coin cells were cycled three times in the voltage range of 1.0–2.5 V, and the corresponding Nyquist plots are shown in Figure 6. It can be seen that each Nyquist plot exhibited a semicircle at high frequency region and a straight line at low frequency region. The surface charge-transfer resistance (*R*_ct_) of NaTi_2_(PO_4_)_3_/C was found to be smaller than that of NaTi_2_(PO_4_)_3_, suggesting that the diffusion of Na^+^ in NaTi_2_(PO_4_)_3_/C is faster than NaTi_2_(PO_4_)_3_ (Lu et al., 2014; Longoni et al., 2016). In addition, the Na^+^ diffusion coefficient (D) can be calculated by the following equations (Ko et al., 2017):

(1) $$\begin{array}{r} \text{D}={\text{R}}^{2}{\text{T}}^{2}/2{\text{A}}^{2}{\text{n}}^{4}{\text{F}}^{4}{\text{C}}^{4}\sigma^{2} \end{array}$$

(2) $$\begin{array}{r} {\text{Z}}^{\text{′}}={\text{R}}_{\text{o}}+{\text{R}}_{\text{ct}}+\sigma \omega^{-0.5} \end{array}$$

in which R is the ideal gas constant, T is the ambient temperature, A is the surface area of the electrode, n is the number of electrons per molecule during intercalation, F is the Faraday constant, C is the concentration of Na^+^ in the active material, σ is the Warburg coefficient, Z' is the real part of the impedance, ω is the angular frequency. The σ value can be calculated by the slope of the plot of *Z'* vs. ω^−0.5^ and presented in Figure S2. The σ value of NaTi_2_(PO_4_)_3_/C was 254 Ω s^−0.5^, much lower than that of NaTi_2_(PO_4_)_3_ (1264 Ω s^−0.5^). Accordingly D of NaTi_2_(PO_4_)_3_/C was larger than that of NaTi_2_(PO_4_)_3_. Summarily, NaTi_2_(PO_4_)_3_/C can effectively restrain the increasing of charge-transfer resistance after multiple discharge and charge cycles, which can improve the rate capability and enhance the cyclic performance at high rate (Song et al., 2014; Roy and Srivastava, 2015).

According to the above results, NaTi_2_(PO_4_)_3_/C has high discharge capacity, good rate capacity, and excellent long-term cycling stability. In addition, compared to other previously reported NaTi_2_(PO_4_)_3_@C composites, the obtained NaTi_2_(PO_4_)_3_/C electrode exhibits excellent properties (Table S1, Supporting Information). The good properties of NaTi_2_(PO_4_)_3_/C could be ascribed to the following reasons: (i) The crystal structure of NASICON-type NaTi_2_(PO_4_)_3_ is an open 3D framework of PO_4_ tetrahedra corner-shared with TiO_6_ octahedra, which can not only provide large spaces for Na^+^ insertion but also supply open tunnels for Na^+^ transport (Boilot et al., 1983; Pang et al., 2014b; Zhao et al., 2015). (ii) The porous structure of nanostructured NaTi_2_(PO_4_)_3_ and carbon matrix can decrease the diffusion length of Na^+^ (Gibaud et al., 1996; Huang et al., 2015; Rui et al., 2016). (iii) The embedding of NaTi_2_(PO_4_)_3_ nanocubes in carbon nanosheets can effectively inhibit the aggregation of the nanocubes, leading to the electrolyte easily penetrating to the active sites.

## Conclusion

In summary, the composition of NaTi_2_(PO_4_)_3_ porous nanocubes and carbon porous nanosheet are successfully developed. The as-obtained NaTi_2_(PO_4_)_3_/C electrodes have good electrochemical properties, including large energy density, excellent rate capacity, and good cycling performance, owing to their special structures and components. The results demonstrate that such NaTi_2_(PO_4_)_3_/C anode is a promising anode for SIBs.

## Author contributions

ZW, XL, CW, and JM design the whole experiment, and write the paper. JL and KF conduct some electrochemical analysis.

### Conflict of interest statement

The authors declare that the research was conducted in the absence of any commercial or financial relationships that could be construed as a potential conflict of interest.
