# Supplementary material for: Porous NaTi2(PO4)3 Nanocubes Anchored on Porous Carbon Nanosheets for High Performance Sodium-Ion Batteries
Source: Front Chem. 2018 Sep 19;6:396. doi: 10.3389/fchem.2018.00396 (PMC6156144; doi:10.3389/fchem.2018.00396)
Supplement: Supplementary file 1 [file Table_1.DOCX]

**Supporting Information**

**Porous NaTi_2_(PO_4_)_3_ nanocubes anchored on porous carbon nanosheets for high performance sodium-ion batteries**

Ziqi Wang,*^a^* Jiaojiao Liang,*^a^* Kai Fan,*^a^* Xiaodi Liu,*^a,c,^** Caiyun Wang*^d,^** and Jianmin Ma*^a,b,^**

*^a^School of Physics and Electronics, Hunan University, Changsha 410022, China*

*^b^Institute of Advanced Electrochemical Energy, Xi’an University of Technology, Xi’an 710048, China*

*^c^College of Chemistry and Pharmaceutical Engineering, Nanyang Normal University, Nanyang 473061, China*

*^d^ARC Centre of Excellence for Electromaterials Science, Intelligent Polymer Research Institute, AIIM Facility, University of Wollongong, North Wollongong, NSW 2500, Australia*

Corresponding authors: X. Liu, C. Wang or J. Ma

liuxiaodiny@126.com (X. Liu)

caiyun@uow.edu.au (C. Wang)

nanoelechem@hnu.edu.cn (J. Ma)


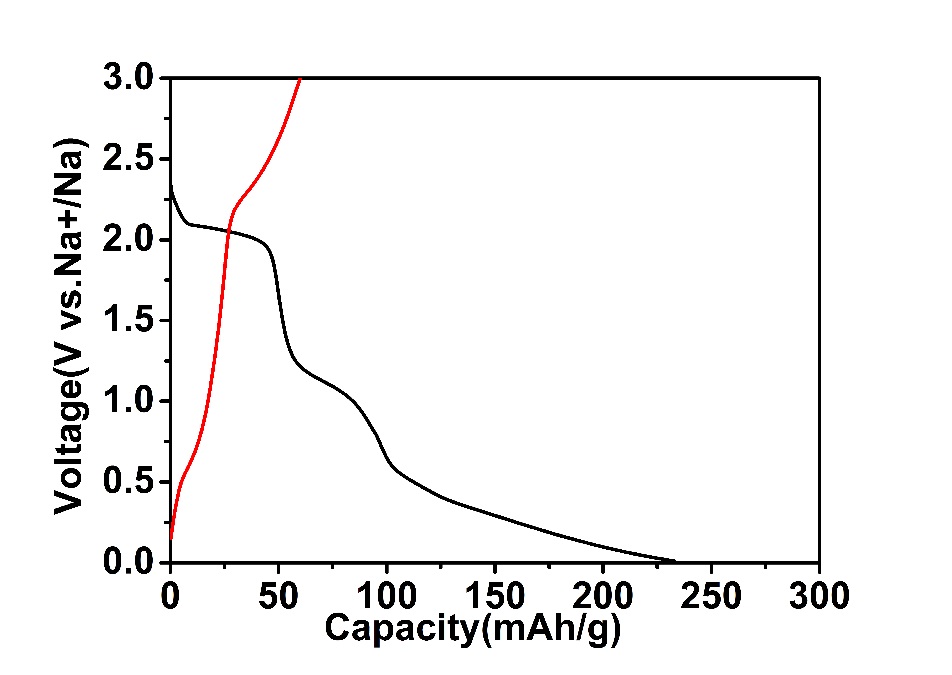


**Fig. S1** The first discharge-charge curve of NaTi_2_(PO_4_)_3_ at a current density of 0.1 A g^-1^

**Tab. 1** Electrochemical properties of NaTi_2_(PO_4_)_3_@C composites as anodes for SIBs

| Materials | Current | Cycle | Capacity  (mAh g^-1^) | Voltage window | Ref. |  |
| --- | --- | --- | --- | --- | --- | --- |
| NTP/C | | 0.5C | 100 | 172 | 0.01-3.0 V | this work |
| NTP@C | | 0.5C | 100 | 201 | 0.01-3.0 V | [[1](#_ENREF_1)] |
| NTP@C | | 0.5C | 350 | 108 | 1.5-3.0 V | [[2](#_ENREF_2)] |
| NTP/C | | 0.5C | 200 | 123 | 1.5-3.0 V | [[3](#_ENREF_3)] |
| NTP @C | | 0.2C | 20 | 111.2 | 1.5-3.0 V | [[4](#_ENREF_4)] |
| NTP /carbon nanofiber | | 0.5 C | 20 | 123.7 | 1.5-3.2 V | [[5](#_ENREF_5)] |
| NTP/N-C | | 0.2C | 30 | 124.2 | 1.5-3.2 V | [[6](#_ENREF_6)] |
| NTP/C nanocomposite | | 1C | 500 | 78.1 | 1.5-3.0 V | [[7](#_ENREF_7)] |
| NTP-TP@rGO nanocomposite | | 1C | 200 | 101 | 1.5-3.0 V | [[8](#_ENREF_8)] |
| NTP/C-NFs | | 2 C | 700 | 110 | 1.5-3.0 V | [[9](#_ENREF_9)] |
| NTP-NCNFs | | 10 C | 2,000 | 121 | 0.01-3.0 V | [[10](#_ENREF_10)] |
| NTP@C nanocomposite | | 0.1C | 30 | 208 | 0.01-3.0 V | [[11](#_ENREF_11)] |
| Carbon-intercalated NTP | | 10C | 100 | 110 | 1.2-2.8 V | [[12](#_ENREF_12)] |
| Carbon-coated NTP | | 1C | 200 | 117 | 1.5-3.0 V | [[13](#_ENREF_13)] |
| NTP /C porous plates | | 1C | 120 | 105 | 1.5-3.3 V | [[14](#_ENREF_14)] |
| NTP/rGO | | 1C | 150 | 100 | 1.0-3.0 V | [[15](#_ENREF_15)] |
| NTP/C-CNTs | | 1C | 200 | 108.5 | 1.5-3.0 V | [[16](#_ENREF_16)] |
| NTP@C@PC | | 1 C | 1000 | 113 | 1.5-2.8 V | [[17](#_ENREF_17)] |
| Mesoporous NTP/CMK-3 | | 0.5 C | 1000 | 62.9 | 1.0-3.0 V | [[18](#_ENREF_18)] |
| Porous NTP-3D graphene | | 1 C | 200 | 101 | 1.5-3.0 V | [[19](#_ENREF_19)] |
| NTP-nanocarbon networks | | 1 C | 800 | 119 | 1.5-2.8 V | [[20](#_ENREF_20)] |

Footnotes: 1C≈200mAh∙g^-1^ (0.01V~3.0V); 1C≈130mAh∙g^-1^ (1.0V~3.0V);

NaTi_2_(PO_4_)_3_: NTP

**Notes and references**

[1] J. Liang, K. Fan, Z. Wei, X. Gao, W. Song, J. Ma, Porous NaTi_2_(PO_4_)_3_@C nanocubes as improved anode for sodium-ion batteries, Mat. Research Bull., 99 (2018) 343-348.

[2] Q. Hu, M. Yu, J. Liao, Z. Wen, C. Chen, Porous carbon-coated NaTi_2_(PO_4_)_3_ with superior rate and low-temperature properties, J. Mater. Chem. A, 6 (2018) 2365–2370.

[3] L. Zhang, X. Wang, W. Deng, X. Zang, C. Liu, C. Li, J.T. Chen, M. Xue, R. Li, F. Pan, Open holey structure enhanced superior rate capability in NaTi_2_(PO_4_)_3_/C nanocomposite for ultralong-life sodium-ion storage, Nanoscale, 10 (2018) 958–963.

[4] X. Yang, K. Wang, X. Wang, G. Chang, S. Sun, Carbon-coated NaTi_2_(PO_4_)_3_ composite: A promising anode material for sodium-ion batteries with superior Na-storage performance, Solid State Ionics, 314 (2018) 61-65.

[5] H. Liu, Y. Liu, 1D mesoporous NaTi_2_(PO_4_)_3_ /carbon nanofiber: The promising anode material for sodium-ion batteries, Ceram. Int., (2017). DOI: https://doi.org/10.1016/j.ceramint.2017.12.147.

[6] D. Xu, P. Wang, R. Yang, Nitrogen-doped carbon decorated NaTi_2_(PO_4_)_3_ composite as an anode for sodium-ion batteries with outstanding electrochemical performance, Ceram. Int., 44 (2018) 7159–7164.

[7] D. Cai, B. Qu, H. Zhan, Porous NaTi_2_(PO_4_)_3_ nanoparticles coated with a thin carbon layer for sodium-ion batteries with enhanced rate and cycling performance, Mater. Lett., 218 (2018) 14-17.

[8] L. Xu, G. Xu, Z. Chen, X. Wei, J. Cao, L. Yang, 3D nanocomposite archiecture constructed by reduced graphene oxide, thermally-treated protein and mesoporous NaTi_2_(PO_4_)_3_ nanocrystals as free-standing electrodes for advanced sodium ion battery, J. Mater. Chem. Sci. Mater. in Electronics, (2018) 1-10.

[9] P. Wei, Y. Liu, Z. Wang, Y. Huang, Y. Jin, Y. Liu, S. Sun, Y. Qiu, J. Peng, Y. Xu, X. Sun, C. Fang, J. Han, Y. Huang, Porous NaTi_2_(PO_4_)_3_/C hierarchical nanofibers for ultrafast electrochemical energy storage, ACS Appl. Mater. Interfaces, (2018). DOI: 10.1021/acsami.8b08415.

[10] S. Yu, Y. Wan, C. Shang, Z. Wang, L. Zhou, J. Zou, H. Cheng, Z. Lu, Ultrafine NaTi_2_(PO_4_)_3_ nanoparticles encapsulated in N-CNFs as ultra-stable electrode for sodium storage, Front. in Chem., 6 (2018). DOI: 10.3389/fchem.2018.00270.

[11] C. Chen, Y. Lu, Y. Ge, J. Zhu, H. Jiang, Y. Li, Y. Hu, X. Zhang, Synthesis of nitrogen-doped electrospun carbon nanofibers as anode material for high-performance sodium-ion batteries, Energy Technol., 4 (2016) 1440-1449.

[12] H. Geng, J. Yang, H. Yu, C. Li, X. Dong, Carbon intercalated porous NaTi_2_(PO_4_)_3_ spheres as high-rate and ultralong-life anodes for rechargeable sodium-ion batteries, Mater. Chem. Front., (2017). DOI: [10.1039/C7QM00048K](https://doi.org/10.1039/C7QM00048K).

[13] C. Xu, Y. Xu, C. Tang, Q. Wei, J. Meng, L. Huang, L. Zhou, G. Zhang, L. He, L. Mai, Carbon-coated hierarchical NaTi_2_(PO_4_)_3_ mesoporous microflowers with superior sodium storage performance, Nano Energy, 28 (2016) 224-231.

[14] Z. Huang, L. Liu, L. Yi, W. Xiao, M. Li, Q. Zhou, G. Guo, X. Chen, H. Shu, X. Yang, Facile solvothermal synthesis of NaTi_2_(PO_4_)_3_/C porous plates as electrode materials for high-performance sodium ion batteries, J. Power Sources, 325 (2016) 474-481.

[15] J. Song, S. Park, J. Gim, V. Mathew, S. Kim, J. Jo, S. Kim, J. Kim, High rate performance of a NaTi_2_(PO_4_)_3_/rGO composite electrode via pyro synthesis for sodium ion batteries, J. Mater. Chem. A, 4 (2016) 7815-7822.

[16] L. Wang, B. Wang, G. Liu, T. Liu, T. Gao, D. Wang, Carbon nanotube decorated NaTi_2_(PO_4_)_3_/C nanocomposite for a high-rate and low-temperature sodium-ion battery anode, RSC Adv., 6 (2016) 70277-70283.

[17] Y. Jiang, L. Zeng, J. Wang, W. Li, F. Pan, Y. Yu, A carbon coated NASICON structure material embedded in porous carbon enabling superior sodium storage performance: NaTi_2_(PO_4_)_3_ as an example, Nanoscale, 7 (2015) 14723-14729.

[18] G. Pang, P. Nie, C. Yuan, L. Shen, X. Zhang, H. Li, C. Zhang, Mesoporous NaTi_2_(PO_4_)_3_/CMK-3 nanohybrid as anode for long-life Na-ion batteries, J. Mater. Chem. A, 2 (2014) 20659-20666.

[19] C. Wu, P. Kopold, Y.-L. Ding, P.A. van Aken, J. Maier, Y. Yu, Synthesizing porous NaTi_2_(PO_4_)_3_ nanoparticles embedded in 3D graphene networks for high-rate and long cycle-life sodium electrodes, ACS nano, 9 (2015) 6610-6618.

[20] Y. Jiang, J. Shi, M. Wang, L. Zeng, L. Gu, Y. Yu, Highly Reversible and ultrafast sodium storage in NaTi_2_(PO_4_)_3_ nanoparticles embedded in nanocarbon networks, ACS Appl. Mater. Interfaces, 8 (2015) 689-695.


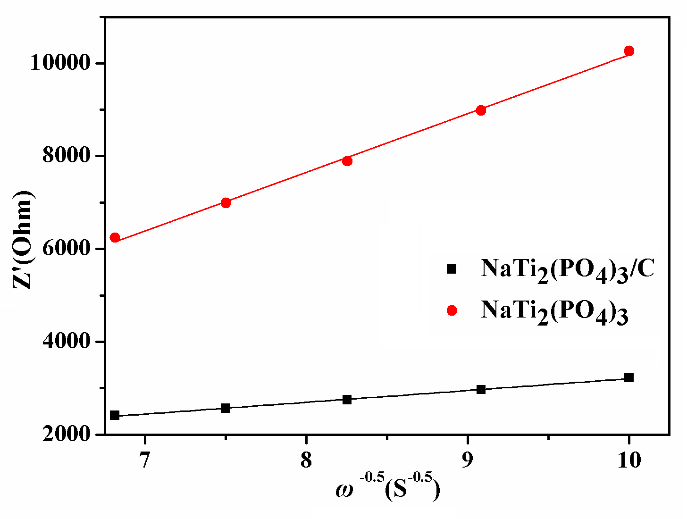


**Fig. S2** Relationship between imaginary resistance (Z’) and inverse square root of angular speed (*ω*^−0.5^) at low frequency region.
